# Estimation of left ventricular activation sequence in patients with heart failure using two-dimensional speckle tracking echocardiography

**DOI:** 10.1007/s10554-023-02834-w

**Published:** 2023-03-27

**Authors:** Hideyuki Hara, Tazuru Igarashi, Toyoji Kaida, Masami Murakami, Hiroshi Ito, Shinichi Niwano, Junya Ako

**Affiliations:** 1 Division of Cardiology, Numazu City Hospital, Aza-Harunoki 550, Higashi-Shiiji, Numazu City, Shizuoka Prefecture 410-0302 Japan; 2 Department of Cardiovascular Medicine, Kitasato University School of Medicine, Sagamihara, Japan

**Keywords:** Longitudinal strain, Time-to-peak strain, Bull’s eye map, Left bundle branch block, Right ventricular pacing, Activation sequence

## Abstract

Evaluation of longitudinal strain (LS) from two-dimensional echocardiography is useful for global and regional left ventricular (LV) dysfunction assessment. We determined whether the LS reflects contraction process in patients with asynchronous LV activation. We studied 144 patients with an ejection fraction ≤ 35%, who had left bundle branch block (LBBB, n = 42), right ventricular apical (RVA) pacing (n = 34), LV basal- or mid-lateral pacing (n = 23), and no conduction block (Narrow-QRS, n = 45). LS distribution maps were constructed using 3 standard apical views. The times from the QRS onset-to-early systolic positive peak (Q-EPpeak) and late systolic negative peak (Q-LNpeak) were measured to determine the beginning and end of contractions in each segment. Negative strain in LBBB initially appeared in the septum and basal-lateral contracted late. In RVA and LV pacing, the contracted area enlarged centrifugally from the pacing site. Narrow-QRS showed few regional differences in strain during the systolic period. The Q-EPpeak and Q-LNpeak exhibited similar sequences characterized by septum to basal-lateral via the apical regions in LBBB, apical to basal regions in RVA pacing, and lateral to a relatively large delayed contracted area between the apical- and basal-septum in LV pacing. Differences in Q-LNpeaks between the apical and basal segments in delayed contracted wall were 107 ± 30 ms in LBBB, 133 ± 46 ms in RVA pacing, and 37 ± 20 ms in LV pacing (*p* < 0.05, between QRS groups). Specific LV contraction processes were demonstrated by evaluating the LS distribution and time-to-peak strain. These evaluations may have potential to estimate the activation sequence in patients with asynchronous LV activation.

## Introduction

Global longitudinal strain from two-dimensional (2D) echocardiography is considered to be an accurate and sensitive parameter for the assessment of global left ventricular (LV) dysfunction [1]. Longitudinal strain (LS) obtained from 3 standard apical views is used for the calculation of global longitudinal strain, but it can also be used to visualize regional dysfunction, such as in myocardial infarction or various types of cardiomyopathy on a bull’s eye map [2]. The LV contraction in left bundle branch block (LBBB) is characterized as an early systolic contraction in the septum followed by delayed contraction in the lateral wall. The mid septum is usually a site of early contraction, while the early contraction is observed in a more apical location during right ventricular apical (RVA) pacing [3]. Such differences in LV contraction are thought to reflect differences in the LV activation sequence between LBBB and RVA pacing [4, 5]. In patients with LBBB or continuous RV pacing, electrical and mechanical dyssynchrony persist; these patients frequently develop heart failure. If the LV activation sequence can be estimated using echocardiography in these patients, additional information relating to the cardiac resynchronization therapy (CRT) response would be available [6, 7]. In the present study, we evaluated the LS during the systolic period and time-to-peak strain using 2D speckle tracking echocardiography and examined the LV contraction process in heart failure patients with LBBB, RVA pacing, and LV pacing.

## Methods

### Patients

Among the patients who underwent echocardiography in our hospital from January 2016 to September 2022, we selected those with LV ejection fraction (LV-EF) ≤ 35%, and with LBBB or a ventricular pacing device (single or dual chamber pacemaker, or CRT). Patients without a conduction block (Narrow-QRS) were also examined. Patients with an irregular RR interval, or in whom the endocardial border was difficult to visualize using echocardiography were excluded. Patients with previous treatment for myocardial infarction or who had undergone coronary artery bypass surgery were excluded. In patients for whom coronary angiography was performed, cases with significant stenosis (≥ 50% or ≥ 75%, respectively, for the left main and other major epicardial arteries) were excluded. After excluding these patients, 144 were enrolled (mean age, 68 ± 12 years; 81 males). Of these patients, coronary angiography was performed in 114 patients (79%). Each patient had one echocardiographic study analyzed. The types of QRS were classified into LBBB (n = 42), RVA pacing (n = 34), LV pacing (n = 23), and no conduction block (Narrow-QRS, n = 45). The criteria for LBBB included a QRS duration ≥ 120 ms, QS or rS in lead V1, broad R waves in leads I, aVL, V5, or V6, and absent Q waves in leads V5 and V6 [8]. The Lead position for RVA pacing was in the apical region of the RV septum [9]. The LV lead position was classified into anterior-lateral (basal, n = 8; mid, n = 4) and inferior-lateral (basal, n = 5; mid, n = 6) [4, 7]. Criteria for the Narrow-QRS were a QRS duration < 120 ms and a normal axis (− 30° to 90°).

### Echocardiography

**Fig. 1:**
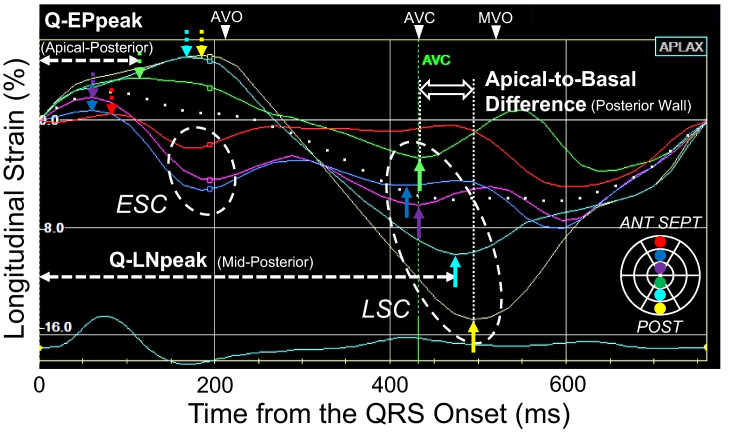
The longitudinal strain (LS) curves in a 3-chamber view of a LBBB patient. Down, dotted arrows indicate the early systolic positive peak (EPpeak). Time from QRS onset-to-EPpeak was measured as the Q-EPpeak. Up arrows indicate the late systolic negative peak (LNpeak) and the time from QRS onset was measured as the Q-LNpeak. The apical-to-basal difference was determined as the time difference in Q-LNpeak between the apical and basal segments. Small rectangles on the strain curve indicate measurement of LS at the end of QRS. *AVO* aortic valve opening, *AVC* aortic valve closing, *MVO* mitral valve opening, *ESC* early systolic contraction, *LSC* late systolic contraction

**Fig. 2:**
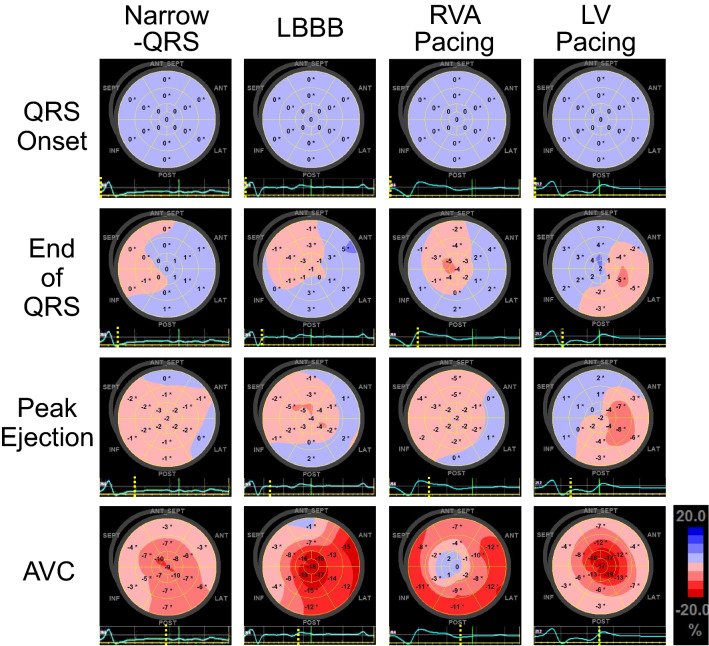
Representative LS distribution during the systolic period. Negative strain indicates contraction, and is displayed in pink or red. The dotted line on the electrocardiogram indicates the time phase of each bull’s eye display. *LBBB* left bundle branch block, *RVA* right ventricular apical, *LV* left ventricle, *Peak Ejection* LV ejection flow showing maximum velocity

All patients underwent echocardiography with a GE Vivid E9 system (GE Vigmed Ultrasound, Horten, Norway). Routine digital grey-scale 2D cineloops were obtained from the 3 standard apical views, and optimized for strain analyses (average 52 frames/s). Quantitative analyses were performed using off-line software (EchoPac, version 113; GE Vigmed Ultrasound). Speckle-tracking LS was evaluated by tracing the endocardial and epicardial borders in 3 apical views. Then, 18 LS curves were obtained in a cardiac cycle [1]. LS was measured every 50 ms from the QRS onset-to-aortic valve closing (AVC), and at the end of QRS (Fig. 1). A bull’s eye map of LS distribution was constructed by measuring the LS in 18 segments (Fig. 2). Although the LV segmentation on echocardiography was based on guidelines [10], we used the six divisions of the LV wall notations used in EchoPac software (in parentheses), as follows: anteroseptal (anterior-septal); inferoseptal (septal); inferior (inferior); inferolateral (posterior); anterolateral (lateral); anterior (anterior). LV volumes were measured in the apical 2- and 4-chamber views and the LV-EF was calculated using the biplane Simpson’s rule. Wall motion score index (WMSI) was visually determined using 17-segment model [11]. Aortic valve opening (AVO) and AVC were determined by the onset and end of pulsed Doppler ejection flow in the LV outflow tract, respectively. Mitral valve opening (MVO) was determined by the onset of the E-wave of transmitral flow.

### Early and late systolic contraction

Early and late systolic contraction were determined from each LS curve (Fig. 1). Early systolic contraction (ESC) was defined as a contraction during the pre-ejection period [12]. Late systolic contraction (LSC) was defined as a contraction that appeared after AVO [13]. If a strain curve indicated ≥ 2 negative peaks, the first peak was selected [13].

### Time-to-peak strain

**Fig. 3:**
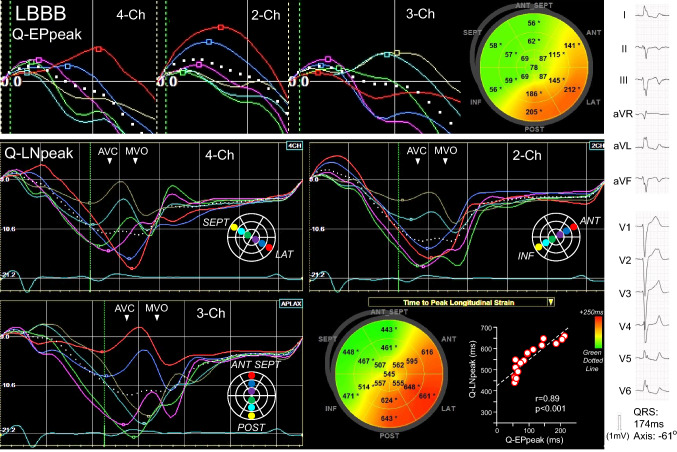
Time-to-peak strain analyses in the LBBB patient presented in Fig. 2. Q-EPpeak and Q-LNpeak in each view prolonged sequentially from the basal or mid segments, apical segments, and to the mid and basal segments in the opposite wall. Q-EPpeaks and Q-LNpeaks showed similar sequences on bull’s eye maps and were positively correlated. Q-EPpeak: time from QRS onset to early systolic positive peak, Q-LNpeak: time from QRS onset to late systolic negative peak. *AVC* aortic valve closing, *MVO* mitral valve opening

**Fig. 4:**
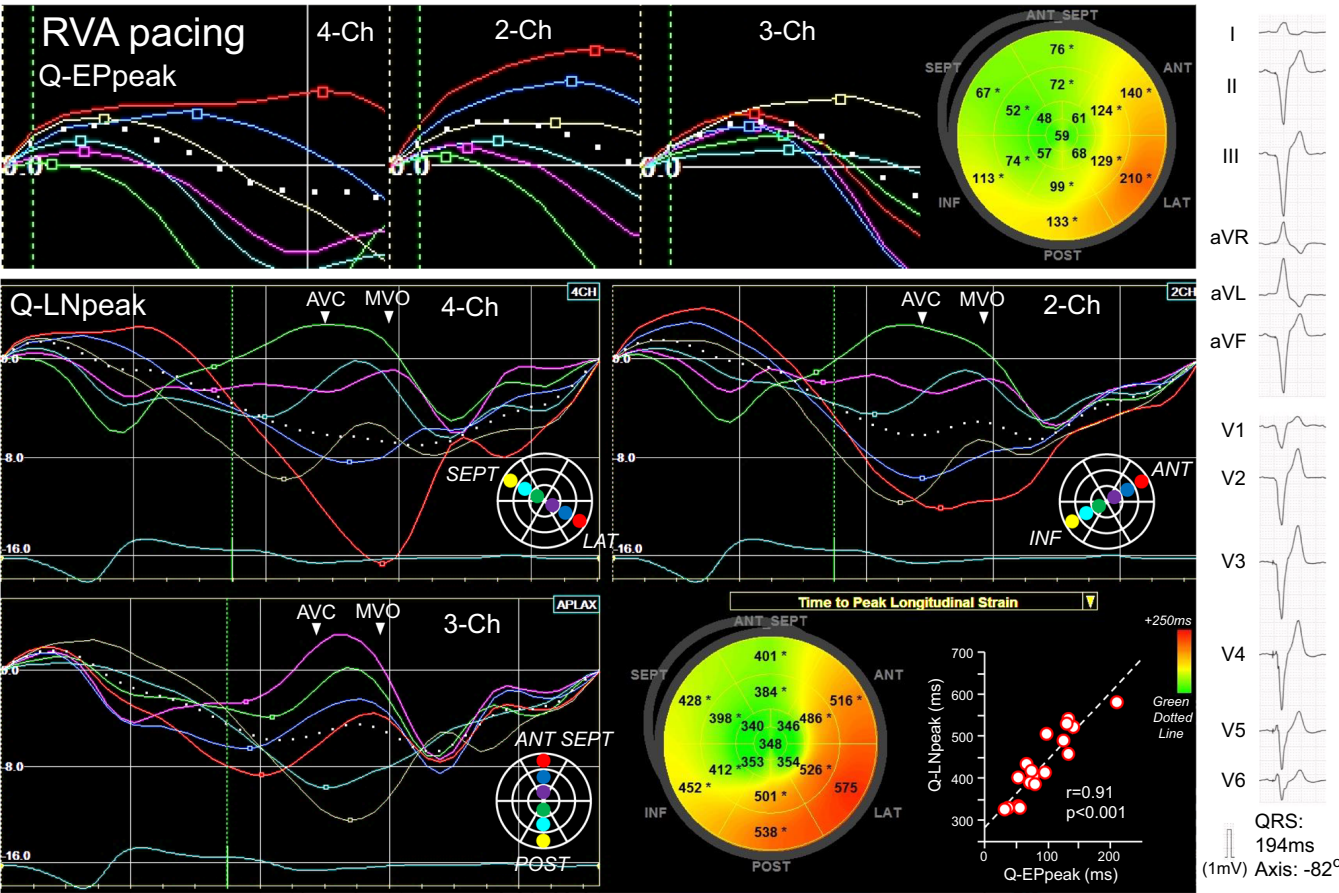
Time-to-peak strain analyses in the patient with RVA pacing presented in Fig. 2. Q-EPpeak and Q-LNpeak prolonged from the apical to basal segments. Q-EPpeaks and Q-LNpeaks showed similar sequences on the bull’s eye maps and were positively correlated

**Fig. 5:**
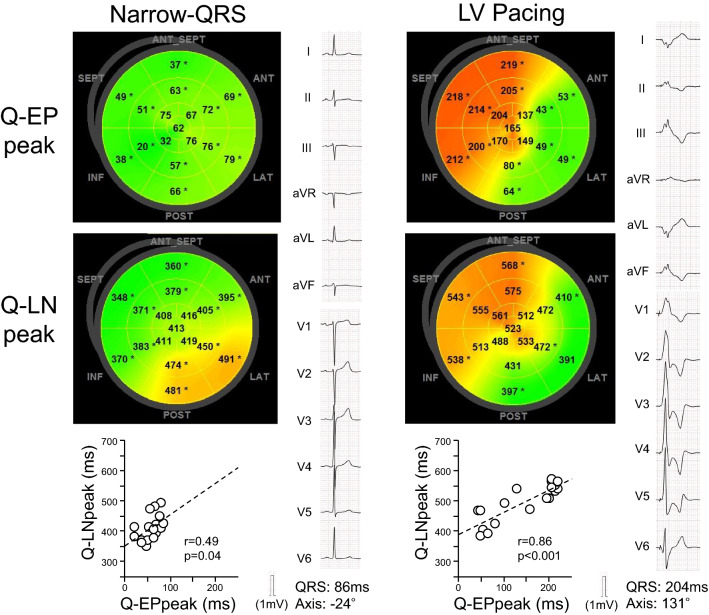
Time-to-peak strain analyses in the patient with Narrow-QRS and the patient with LV pacing presented in Fig. 2. The patient with Narrow-QRS did not show a specific contraction sequence. In the patient with LV pacing, Q-EPpeaks and Q-LNpeaks were short around the lateral wall and prolonged in the septal and anterior-septal walls

The early systolic positive peak (EPpeak) was defined as a positive peak appearing after QRS onset [14]. The time from QRS onset-to-EPpeak was measured as the Q-EPpeak (Fig. 1). If the strain curve became negative after QRS onset, the Q-EPpeak was 0 ms (77 of 2592 segments [3.0%]). The late systolic negative peak (LNpeak) was defined as the LSC peak. The time from QRS onset-to-LNpeak was measured as the Q-LNpeak. The LNpeak was determined automatically by the software, but in some cases that needed correction and the EPpeak were determined manually by moving the marker on the strain curve. Bull’s eye maps of the Q-EPpeak and Q-LNpeak were constructed by measuring the peaks on 18 strain curves (Figs. 3, 4, 5).

### Apical-to-basal difference

**Fig. 6:**
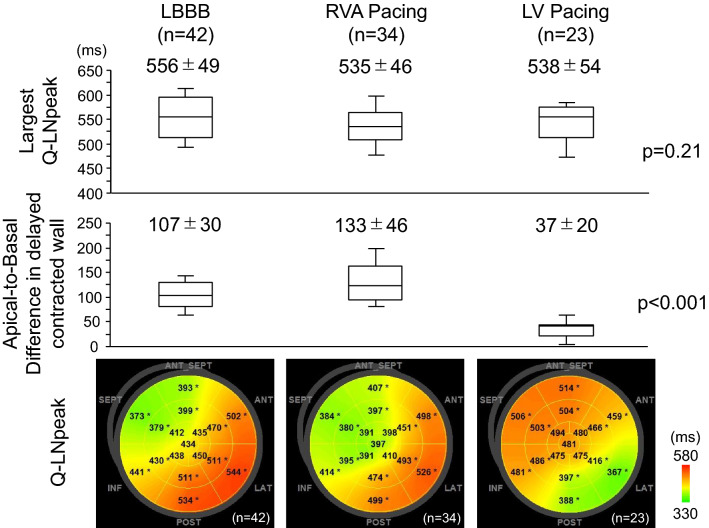
Largest Q-LNpeak in 18 segments (upper panel), apical-to-basal difference in the delayed contracted wall (middle panel) and segmental Q-LNpeak (lower panel). The largest Q-LN-peak was not different in patients with LBBB, RVA pacing, and LV pacing. The apical-to-basal difference was smallest in LV pacing, followed by LBBB and RVA pacing with significant differences between groups. The delayed contracted wall (including the segment with the largest Q-LNpeak in 18 segments) was determined in the posterior (n = 12), lateral (n = 29) and anterior walls (n = 1) in LBBB, posterior (n = 5), lateral (n = 19) and anterior walls (n = 10) in RVA pacing, and anterior-septal (n = 15) and septal walls (n = 8) in LV pacing

Differences in Q-LNpeaks between the apical and basal segments (apical-to-basal difference) were calculated in each division of the LV walls (anterior-septal, septal, inferior, posterior, lateral, and anterior walls). This was done by subtracting the minimum from the maximum Q-LNpeak in three segments (apical, mid and basal segments; Fig. 1). The apical-to-basal difference in delayed contracted wall (a wall that includes the largest Q-LNpeak in 18 segments) was compared in patients with LBBB, RVA pacing, and LV pacing (Fig. 6).

### Mid-apical-opposite wall sequence

In patients with LBBB, the Q-LNpeaks in the 4- and 3-chamber views usually prolonged sequentially from the mid or basal septal (anterior-septal), apical segments, and to the mid and basal lateral (posterior) (Fig. 3). If such a Q-LNpeak sequence (i.e., prolonged from mid or basal segment, apical segments, and to the opposite wall) was observed in a view with an apical-to-basal difference ≥ 60 ms in the opposite wall, the sequence of the Q-LNpeak in that view was regarded as showing the mid-apical-opposite wall sequence. We evaluated how many of 3 views indicate the mid-apical-opposite wall sequence (Table 1).

**Table 1:** Patient characteristics and longitudinal strain evaluations

	Narrow-QRS	LBBB	RVA pacing	LV pacing	*p*
	n = 45	n = 42	n = 34	n = 23	
Age (years)	64 ± 15	69 ± 9	70 ± 10	68 ± 7	0.08
Male (n)	27 (60%)	20 (48%)	22 (65%)	12 (52%)	0.45
Hypertension (n)	26 (58%)	24 (57%)	22 (65%)	14 (61%)	0.91
Diabetes mellitus (n)	5 (11%)	10 (24%)	8 (24%)	6 (26%)	0.34
Creatinine > 1.5 mg/dl (n)	5 (11%)	6 (14%)	10 (29%)	7 (30%)	0.08
Medications					
ACE inhibitor or ARB (n)	42 (93%)	32 (76%)	27 (79%)	20 (87%)	0.14
Beta-blocker (n)	39 (87%)	28 (67%)	24 (71%)	21 (91%)	0.04
Spironolactone (n)	25 (56%)	17 (40%)	10 (29%)	9 (39%)	0.13
Amiodalone (n)	1 (2%)	1 (2%)	2 (6%)	3 (13%)	0.20
NYHA class III/IV (n)	5 (11%) / 1 (2%)	5 (12%) / 0 (0%)	4 (12%) / 0 (0%)	4 (17%) / 0 (0%)	0.83
Baseline parameters					
QRS duration (ms)	101 ± 10	151 ± 17^†^	174 ± 24^†‡^	184 ± 21^†‡^	< 0.001
QRS axis (degree)	32 ± 34	− 22 ± 37^†^	− 72 ± 10^†‡^	189 ± 55^†‡§^	< 0.001
Heart rate (beats/min)	72 ± 14	70 ± 13	77 ± 14	74 ± 12	0.14
EDV (ml)	121 ± 38	121 ± 50	114 ± 44	129 ± 60	0.71
ESV (ml)	86 ± 29	87 ± 37	83 ± 36	95 ± 50	0.68
LV-EF (%)	29 ± 4	28 ± 4	28 ± 5	29 ± 7	0.79
LV diameter diastolic (mm)	57 ± 8	56 ± 8	55 ± 8	56 ± 11	0.62
IVS thickness (mm)	11 ± 2	10 ± 2	10 ± 2	10 ± 2	0.29
PW thickness (mm)	11 ± 2	11 ± 2	12 ± 2	12 ± 2	0.18
LA diameter (mm)	38 ± 7	38 ± 8	41 ± 8	37 ± 8	0.34
WMSI	2.3 ± 0.1	2.3 ± 0.2	2.4 ± 0.2	2.4 ± 0.3	0.06
Longitudinal strain evaluations					
SD of strain at the end of QRS (%)	1.4 ± 0.6	3.0 ± 0.9^†^	2.9 ± 1.2^†^	3.1 ± 1.2^†^	< 0.001
Segments indicating ESC (segments)	1.5 ± 2.2	4.6 ± 2.2^†^	4.5 ± 2.6^†^	1.7 ± 1.3^‡§^	< 0.001
Patients with at least one ESC (n)	20 (44%)	39 (93%)	31 (91%)	18 (78%)	< 0.001
ESC in AS/S/I/P/L/A walls (segments)	0.3/0.4/0.4/0.1/0.2/0.2	1.5/2.1/0.8/0/0/0	0.9/1.8/1.1/0.2/0.1/0.3	0/0/0/0.7/0.7/0.3	
ESC in basal/mid/apical (segments)	0.5/0.4/0.5	1.4/1.6/1.6	0.8/1.5/2.4	1.1/0.3/0	
Mid-apical-opposite wall sequence (views)	0.6 ± 0.8	2.1 ± 0.7^†^	0.6 ± 0.8^‡^	0.2 ± 0.4^‡^	< 0.001
Largest Q-EPpeak (ms)	166 ± 50	196 ± 35^†^	198 ± 53^†^	214 ± 29^†^	< 0.001
Largest Q-LNpeak (ms)	498 ± 48	556 ± 49^†^	535 ± 46^†^	538 ± 54^†^	< 0.001
Apical-to-basal difference (ms)	91 ± 48	107 ± 30	133 ± 46^†‡^	37 ± 20^†‡§^	< 0.001
Correlation of Q-EPpeak and Q-LNpeak (r)	0.28 ± 0.36	0.75 ± 0.15^†^	0.71 ± 0.22^†^	0.63 ± 0.27^†^	< 0.001

*LBBB* left bundle branch block, *RVA* right ventricular apical, *LV* left ventricle, *ACE* angiotensin converting enzyme, *ARB* angiotensin II receptor blocker, *NYHA* New York Heart Association, *EDV* end diastolic volume, *ESV* end systolic volume, *LV-EF* left ventricular ejection fraction, *IVS* intraventricular septum, *PW* posterior wall, *LA* left atrial, *WMSI* wall motion score index, *SD* standard deviation, *ESC* early systolic contraction, *AS* anterior-septal, *S* septal, *I* inferior, *P* posterior, *L* lateral, *A* anterior, *Mid-apical-opposite wall sequence* sequence of Q-LNpeak prolonged sequentially from the mid or basal segment, apical segments, and to the opposite wall, *Q-EPpeak* time from QRS onset to early systolic positive peak, *Q-LNpeak* time from QRS onset to late systolic negative peak, *Apical-to-basal difference* differences in Q-LNpeaks between the apical and basal segments in delayed contracted wall

^†^*p* < 0.05 versus narrow QRS, ^‡^*p* < 0.05 versus LBBB and ^§^*p* < 0.05 versus RVA pacing in Tukey–Kramer *post-hoc* test

### Early and late contraction sites

**Fig. 7:**
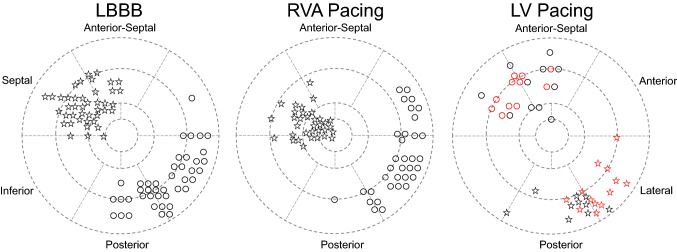
The early contraction site (asterisks) and late contraction site (circles) are shown. In LV pacing, patients paced from the anterior-lateral are shown in red, and patients paced from the inferior-lateral are shown in black

In patients with LBBB, RVA pacing, and LV pacing, the site of early contraction was determined as the center of the negative strain area at the end of QRS (Fig. 7). The site of late contraction was determined as the segment which had the largest Q-LNpeak in 18 segments. If there were segments that had a Q-LNpeak within 20 ms of the largest Q-LNpeak, the late contraction site was marked between these segments and the segment with the largest Q-LNpeak (Fig. 7).

### Statistical analyses

The data are presented as a mean ± standard deviation (SD). Categorical parameters were compared using chi-square analysis followed by Fisher’s exact test. Differences between QRS groups in continuous variables were evaluated using ANOVA with the Tukey–Kramer *post-hoc* test. The relationship between the Q-EPpeak and Q-LNpeak was determined using linear regression analysis. A *p* value < 0.05 was considered statistically significant. Intra- and inter-observer variabilities of time-to-peak LS (Q-EPpeak and Q-LN peak) were assessed in 25 randomly selected patients (6 patients with Narrow QRS, 8 patients with LBBB, 6 patients with RVA pacing and 5 patients with LV pacing). Intra-observer variability was assessed by H.H. Inter-observer variability was assessed by H.I. who is blind to the QRS morphology. Variability was expressed as the absolute difference, absolute difference divided by the mean value, and the intra-class correlation coefficient (ICC).

## Results

### Longitudinal strain distributions during the systolic period

Table 1 shows the patient characteristics in each QRS group. Age or gender, and number of patients with hypertension, diabetes mellitus or impaired renal function were not different between the QRS groups. There were no differences in LV chamber size, LV-EF, wall thickness, or WMSI between the QRS groups. No differences were found for medications (except for beta-blockers) or for heart failure symptoms. Representative LS distributions during the systolic period are shown in Fig. 2. In a patient with LBBB, negative strain appeared in the mid septum. The contracted area enlarged including the apical regions, and the basal lateral region contracted late. A patient with Narrow-QRS exhibited few regional differences in strain at the end of QRS. Contractions continued during the systolic period in each segment that appeared to be synchronously contracted. The SD of the segmental LS at the end of QRS was significantly smaller compared to the other QRS groups (Table 1). In a patient with RVA pacing, the early contraction appeared in the apical septal, and the contracted area enlarged toward the basal regions. In a patient with LV pacing, the early contraction appeared in the mid lateral, and the contracted area enlarged toward the remote regions.

### Prevalence of early and late systolic contraction

Compared to the patients with a Narrow-QRS, patients with LBBB or RVA pacing had a larger number of segments with ESC (Table 1). In patients with LBBB or RVA pacing, ESC was mainly observed in the anterior-septal, septal, or inferior walls. In patients with RVA pacing, approximately one-half of the segments with ESC were in the apical segments. Patients with LV pacing had segments with ESC mainly in the lateral and posterior walls, and there were no apical segments with ESC. Mid-apical-opposite wall sequence was most frequently observed in LBBB compared to other QRS groups. In the 144 patients enrolled in this study, LSC was identified in 2347 of 2592 segments (91%). In the 449 segments with ESC, 357 segments (80%) were accompanied by LSC.

### Segmental time-to-peak strain

Figures 3, 4 and 5 show the time-to-peak strain analyses in patients presented in Fig. 2. In the patient with LBBB, the Q-EPpeak and Q-LNpeak in each view prolonged sequentially from the mid or basal segments, apical segments, and to the mid and basal segments in the opposite wall (Fig. 3). The Q-EPpeak and Q-LNpeak showed similar sequences on the bull’s eye maps. A positive correlation was observed between the Q-EPpeak and Q-LNpeak. In the patient with RVA pacing, the Q-EPpeak and Q-LNpeak in each view were short in the apical segments and prolonged to the basal segments (Fig. 4). The Q-EPpeak and Q-LNpeak had similar sequences and were positively correlated. In the patient with Narrow-QRS, it was difficult to identify a specific Q-EPpeak and Q-LNpeak sequence (Fig. 5). The correlation between the Q-EPpeak and Q-LNpeak was weak (r = 0.49, *p* = 0.04). In the patient with LV pacing, the Q-EPpeak and Q-LNpeak were short around the lateral wall, and prolonged in the septal and anterior-septal walls (Fig. 5). Among the patients with LBBB, RVA pacing, and LV pacing, the apical-to-basal difference in the delayed contracted wall was smallest in patients with LV pacing, followed by those with LBBB and RVA pacing (*p* < 0.05, between QRS groups; Fig. 6). Intra-observer variability for determining Q-EPpeak was 19 ± 20 ms (23 ± 33%) with an ICC of 0.90, and inter-observer variability was 25 ± 33 ms (29 ± 48%) and ICC of 0.79. Intra-observer variability for Q-LNpeak was 20 ± 27 ms (4 ± 6%) and ICC of 0.89, and inter-observer variability was 30 ± 40 ms (7 ± 9%) and ICC of 0.77.

### Early and late contraction sites

The distribution of early and late contraction sites was relatively similar for LBBB and RVA pacing (Fig. 7). However, RVA pacing tended to have more early contraction sites in the apical region. The late contraction sites in RVA pacing were more common in the basal segments compared to LBBB (25/34 [74%] vs. 20/42 [48%], *p* = 0.02). The late contraction sites in LV pacing scattered around the mid region.

## Discussion

The contraction sequence of the entire LV has been investigated using tagged MRI [15, 16], SPECT [17], electro-anatomical mapping (NOGA) [18], and 3D echocardiography [19]. 2D speckle tracking echocardiography has been used to evaluate the LV contraction abnormalities, but to our knowledge, this is the first report to evaluate the LV contraction process by constructing bull’s eye maps.

The LV contraction process in LBBB is thought to be caused by abnormalities in LV activation [20, 21]. Based on electro-anatomical mapping using unipolar electrodes, a U-shaped activation wave front was observed moving from the LV breakthrough site to the lateral wall turning around the apical regions [20]. In our study, LBBB patients showed the majority of the ESC in the septal and anterior-septal walls (Table 1). The mid-apical-opposite wall sequence was observed in both 4- and 3-chamber views in 74% of the patients with LBBB. These results are thought to reflect the LV activation abnormalities in LBBB.

The LV activation sequence in RVA pacing has been reported to differ from that in LBBB [4, 5]. Using electro-anatomical mapping, Mafi Rad et al. evaluated the LV activation sequence in CRT candidates [4]. At the time of RVA pacing, the LV was activated in the apical-to-basal direction and the regions of late activation shifted more toward the basal regions compared to the time of LBBB. In our study, ESC was observed most frequently in the apical segments in patients with RVA pacing (Table 1), and the LV contraction tended to proceed from apical to basal directions (Fig. 4). Such LV contraction processes observed in RVA pacing are thought to be due to the LV activation initiated from the apical regions.

Miranda et al. evaluated the timing of RV activation at the time of LV pacing [22]. Electrical separation from the LV activation to the RV mid septum was 161.2 ± 23.7 ms, which was larger when compared to the RV outflow tract (154.1 ± 20.8 ms) or RV apex (148.0 ± 25.5 ms). The largest electrical separation was most frequently observed in the mid septum (40 of 50 patients). In our study, late contraction sites in patients with LV pacing were mainly observed in the mid segments in the septum (septal or anterior-septal wall; Fig. 7). This result did not contradict the reports of Miranda et al. [22]. The apical-to-basal difference in delayed contracted wall was smaller for LV pacing compared to LBBB and RVA pacing (Fig. 6). Assuming that the Q-LNpeak in a segment reflects timing of LV activation in that segment [13], our results indirectly indicate that the timing of LV activation between the apical and basal segments in the septum did not differ significantly at the time of LV pacing. In several reports that compared the response to CRT between patients with RVA and non-apical pacing, clear differences were not observed [23–26]. This result may be related to the activation delay feature in the RV septum at the time of LV pacing.

There are few reports evaluating the beginning of contraction in a segment to estimate the LV activation sequence [15]. Echocardiography has not been used extensively for this purpose due to the difficulty in evaluating subtle changes in myocardial shortening during early systole. Seo et al. [19] evaluated regional deformation (area change ratio) using three-dimensional (3D) echocardiography, and analyzed the timing of area change ratio, which indicated 25% of the maximum value. Seo et al. observed a U-shaped propagation of the LV contraction in patients with typical LBBB. In our study, the Q-EPpeak had similar sequences to the Q-LNpeak in patients with LBBB, RVA pacing, and LV pacing. Therefore, the Q-EPpeak also has the ability to reflect the LV activation sequence. We think strain analysis is a sensitive and useful tool for the assessment of ventricular contraction process, and future studies are expected to investigate its relevance to the electrophysiological findings. Such studies could potentially enable non-invasive prediction of CRT effects and selection of optimal lead location using echocardiography.

## Study limitations

First, evaluation using the apical images in 2D echocardiography did not provide information on circumferential directions. There was a large spatial gap, especially in the basal regions, and this might have reduced the number of segments with ESC in LV pacing (Table 1). Three-dimensional echocardiography obtains information from the entire LV and evaluates deformation in a local segment [27]. Although there is a need to improve temporal and spatial resolutions, 3D echocardiography is thought to be a useful tool that improves on 2D echocardiography. For time-to-peak strain analysis, it depends on temporal resolution. It was difficult to identify the earliest contraction site using Q-EPpeak, as in the identification of the earliest excitation site in the electrophysiological testing. Second, the present study did not examine patient prognosis or the relationship to CRT efficacy. Although there were reports of latest contraction site and CRT efficacy assessed using radial strain [6, 7], the clinical usefulness of assessing longitudinal strain from the three apical views is unclear. Third, since the patients in each QRS group were different, differences in the results between the QRS groups cannot be considered as a change. Mafi Rad et al. [4] and Jackson et al. [5] examined differences during LBBB and RVA pacing within the same patient, allowing the change to be evaluated. In order to perform an evaluation such as change in time-to-peak strain in a segment when switching from LBBB to RVA pacing, it is necessary to study this within the same patient. Finally, the time-to-peak strain may be influenced by factors other than electrical activation, such as scar formation or load to the myocardium [15, 28]. These factors were not clear in this study.

## Conclusion

Evaluation of the LS distribution during the systolic period and the time-to-peak strain using 2D speckle tracking echocardiography are thought to be useful for identifying the LV contraction process in patients with an asynchronous LV activation sequence. The sites of early contraction and subsequent LV contraction were visualized using bull’s eye LS distribution maps. The time-to-peak strain could be used to determine the intersegmental contraction sequence and late contraction site.
